# Supplementary material for: Multi-organ structural and functional deficits in association with long COVID: a population-based case-control study
Source: eClinicalMedicine. 2026 Jul 23;98:104104. doi: 10.1016/j.eclinm.2026.104104 (PMC13427505; doi:10.1016/j.eclinm.2026.104104)

Supplementary table S1 - Symptoms elicited at clinic (in alphabetical order).

Arrhythmias/palpitations

Chest pain

Confusion/difficulties concentrating

Conjunctivitis/sore eye

Cough

Fatigue

Headache

Joint pain

Loss of smell

Loss of taste

Low grade fever

Muscle aches

Nausea/lack of appetite

Other

Runny nose

Shortness of Breath

Sore throat

Vertigo

Supplementary table S2. Items comprising the nine health domains

DOMAINS		SELECTED VARIABLE(S)				DERIVED VARIABLES			DOMAIN-SPECIFIC SCORE
**Health domain**	**Sub-domain**	**Trait**	**Variable/s**	**Measure/s**	**Number of times measured**	**Derived Metric/s [item coding]**	**Criteria used to define thresholds; thresholds (if relevant)**	**Reference Paper (if relevant)**	**0 – no deficit, 1 – mild deficits; 2 – moderate deficits; 3 – severe deficits**
**1) Autonomic function**	**Postural blood pressure (BP)**	Orthostatic hypotension	Change in peripheral BP from lying to standing after 1min and 3min	Lying systolic BP (SBP) / diastolic BP (DBP)<br>Standing SBP/DBP	3 (repeated)<br>2 (2 timepoints)	Orthostatic hypotension (Change in peripheral BP from lying [average of 2nd and 3rd measures] to standing after 1min or 3min)<br>[0/1]	Guidelines;<br>Orthostatic hypotension: decrease in SBP by ≥20 mmHg, or decrease in DBP by ≥10 mmHg, within 3min after standing<br>(change ≤ -20 SBP / -10 DBP at 1 min or at 3 min coded as 1)	https://academic.oup.com/bmb/article/115/1/123/260117	0: No deficits<br>1: deficit in 1 metric<br>2: deficits in 2 metrics<br>3: deficits in at least 3 metrics
	**Heart rate**	Sinus tachycardia	Resting heart rate while seated (HR)	Seated HR	3	Sinus tachycardia [0/1]	Guidelines; Mean of three seated HR >100 = 1	2015 ACC/AHA/HRS Guideline for the Management of Adult Patients With Supraventricular Tachycardia | Circulation	
		Dysfunctional heart rate variability (HRV)	Time and frequency metrics on HR profiles	Root mean square of successive differences between normal-to-normal intervals (RMSSD)	1	HRV abnormality [0/1]	Literature; low RMSSD <25 ms	https://pubmed.ncbi.nlm.nih.gov/20663071/; ps://pubmed.ncbi.nlm.nih.gov/20663071/;<br>https://www.frontiersin.org/articles/10.3389/fpubh.2017.00258/full	
		Dysfunctional heart rate recovery (HRR)	Change from maximum HR to 1min after stopping the CPET test	Change from maximum HR to 1min after stopping the CPET test	1	HRR abnormality [0/1]	Guidelines;<br>HRR ≤25 bpm = 1	https://www.nejm.org/doi/full/10.1056/nejmoa043012	
		Postural tachycardia	Change in HR from lying to standing after 1min and 3min	Lying HR<br>Standing HR	3 (repeated)<br>2 (2 timepoints)	POTS<br>[0/1]	Guidelines;<br>Postural tachycardia: Increase in HR by ≥30 bpm, within 3min after standing<br>(change ≥30 at 1 min or at 3 min coded as 1)	https://www.ncbi.nlm.nih.gov/pmc/articles/PMC9012474/	
**2) Vascular**	**Resting Blood pressure**	Seating peripheral BP	Average peripheral BP	Seating peripheral SBP/DBP	3	Hypertension groups (mean of 2nd and 3rd BP measures) [0/1/2/3]	Guidelines; ≥140 SBP / 90 DBP = 3; 130-140/80-90 = 2; 120-130 & <80 = 1; <120 = 0	https://www.heart.org/en/health-topics/high-blood-pressure/understanding-blood-pressure-readings; https://www.escardio.org/Journals/E-Journal-of-Cardiology-Practice/Volume-17/definition-of-hypertension-and-pressure-goals-during-treatment-esc-esh-guidelin	0: history=0 & hypertension ranges=normal<br>1: history=0 & hypertension ranges=mild<br>2: history=0 & hypertension ranges=moderate<br>3: history=1 or hypertension ranges=severe
		Hypertension<br>Hypertensive medication	Self-reported hypertension<br>Self-reported hypertensive medication	Hypertension<br>Use of antihypertensive	1<br>1	History of hypertension (history or medication) [0/1]			
**3) Exercise Response**	**Cardiopulmonary exercise test (CPET)**	% Predicted peak VO2	Predicted VO2 max achieved using age, sex, weight and height	Predicted peak VO2	1	Predicted peak VO2<br>[coded 0/1/2/3]	Guidelines;<br>0: >84% ; 1: >70%, ≤84%; 2: >50%, ≤70%; 3: ≤50% or meeting exclusion criteria for CPET	https://respiratory-research.biomedcentral.com/articles/10.1186/s12931-021-01895-6	Scored as per predicted peak V02 categories
**4) Muscle strength**	**Bioimpedance**	Low percentage muscle mass (PMM)		TABC_PMM		Low muscle mass<br>[0/1]	PMM <40% = 1	https://www.ncbi.nlm.nih.gov/pmc/articles/PMC8350199/	0: no deficits; 1: deficit in one item; 3; deficit in both item (NB: omits second category)
	**Hand-grip strength**	Hand-grip strength		Hand-grip strength	3	Hand-grip strength based on maximum test value of three<br>[0/1]	Literature; Sex- and age-dependent cut-offs for low grip strength (<10th percentile) = 1	https://journals.plos.org/plosone/article?id=10.1371/journal.pone.0113637	
**5) Physical function**	**Sit-to-stand test**	FTSTS or 5xSTS (five times sit to stand test)<br>TTSTS or 10xSTS (ten times sit to stand test)	Time to complete up to 10 chair rises (also available time for 5)	chair rises	1	Chair rises<br>[0/1]	Literature; age-dependent cut-offs (age<70, 5 rises in ≥11.4 seconds =1; age 70-79, 5 rises in ≥12.6 seconds =1; age≥80, 5 rises in ≥14.8 seconds = 1)	https://pubmed.ncbi.nlm.nih.gov/17037663/<br>https://journals.lww.com/nsca-jscr/Fulltext/2011/11000/Test_Retest_Reliability_of_the_Five_Repetition.36.aspx	0: no deficits; 1: deficit in one item; 3; deficit in both item (NB: omits second category)
	**Balance exercises**	Flamingo balance exercises	Flamingo balance exercises: time for balance with one leg raised with eyes open and closed	Flamingo balance exercises	eyes open: 2 per leg<br>eyes closed: 1 per leg	Balance<br>[0/1]	Literature; age-dependent cut-offs; performance with open eyes<15 seconds or performance with closed eyes <5 seconds = 1	https://pubmed.ncbi.nlm.nih.gov/19839175/	
**6) Brain**	**Brain MRI**	Incidental findings		Incidental findings	1	Incidental findings [0/1]			Domain score = highest score between WBV and WMHV items; anyone with incidental findings coded 3 irrespective of WBV and [illegible]
		Whole brain volume (WBV)		WBV		Whole brain volume<br>[0/1/2/3]	Data-driven; Based on distributions of this variable in control group 3 (no report of COVID-19 from recruitment) without comorbidities, thresholds at <10th centile [1], <5th centile [2] or <1st centile [3]	https://pubmed.ncbi.nlm.nih.gov/19839175/l29 [illegible]	

		Cerebrovascular lesions		Total white matter hyperintensities		White matter hyperintensities [0/1/2/3]	Data-driven; Based on distributions of this variable in control group 3 (no report of COVID-19 from recruitment) without comorbidities, thresholds at >90th centile [1], >95th centile [2] or >99th centile [3]	[illegible]	findings coded 0 irrespective of WBV and WMHV scores
7) Heart	Cardiac MRI	Incidental findings		Incidental findings	1	Incidental findings [0/1/3]	Anyone with advisory CV-related note = 1; anyone with urgent CV-related note = 3	NA	0: no deficits or incidental findings; 1: advisory incidental findings only; 2: 1 deficit across items, but no advisory findings; 3: 2 or more deficits across items, or urgent incidental finding
		T1 (longitudinal magnetization relaxation time constant)		T1 Global		T1 Global outlier [0/1]	Data-driven; 1 = participants >2 standard deviations from mean based on distributions of these variables in control group 3 (no report of COVID-19 from recruitment) without comorbidities		
		T2 ((transverse magnetization relaxation time constant) - global and middle		T2 Global		T2 Global outlier [0/1]			
		Late gadolinium enhancement (LGE)		Late gadolinium enhancement (LGE)		LGE [0/1]	Any enhancement classified as abnormal		
		Left & right ventricular ejection fractions (R/LVEF)		Left & right ventricular ejection fractions (R/LVEF)		EF [0/1]	LVEF<50 = 1, RVEF<35 = 1	https://www.sciencedirect.com/science/article/pii/S1097664723010529?via%3Dihub	
	Resting ECG	ECG abnormalities		ECG abnormalities	1	Incidental findings [0/1]	Any participant with an advisory note from clinical review = 1	NA	
8) Pulmonary	Spirometry	Forced Expiratory Volume in the 1st second (FEV1)<br>Forced Vital Capacity (FVC)	Externally derived z-scores for deviations from age- and sex-specific reference values from the GLI (https://gli-calculator.ersnet.org/index.html)	Z-FEV1 against external reference ranges<br>Z-FVC against external reference ranges<br>Z-FEV1/FVC ratio against external reference ranges	1	FEV1 [0/1]<br>FVC [0/1]	For each of z-FEV1, z-FVC, zFEV1/FVC: 0= measure ≥-1.645; 1= measure <-1.625 & measure ≥-2; 2 = measure <-2 & measure ≥-3; 3 = measure <-3	https://pubmed.ncbi.nlm.nih.gov/22743675/	Domain score = highest score from zFEV1, zFVC and zFEV1/FVC categories
	Lung MRI	Lung parenchymal abnormality	Qualitative axial thoracic HASTE images	four categories; up to 25%, 26-50%, 51%-75% and 76-100%	1	0-4		https://doi.org/10.1016/s2213-2600(23)00262-x	
9) Renal	Renal MRI	Renal corticomedullary differentiation	Difference in the visualization of cortex and medulla; abnormality can indicate nephropathy	Renal corticomedullary differentiation	1	CMD in the left and right kidneys (mean)	Data-driven; Based on distributions of this variable in control group 3 (no report of COVID-19 from recruitment) without comorbidities, thresholds at <10th centile [1], <5th centile [2] or <1st centile [3]	NA	Domain score = highest score between CMD and eGFR categories
	Renal function	eGFR		eGFR	1		0 = eGFR ≥60 ; 1 = eGFR ≥45 & eGFR<60, 2 = eGFR ≥30 & eGFR<45, 3 = eGFR<30	https://www.sciencedirect.com/journal/kidney-international-supplements/vol/3/issue/1	

Supplementary table S3 – Overall median deficit score, and domain specific presence or absence of deficit by case and control subgroup status

Outcome measure	N (non-missing)	Case-control group			
		Cases N = 141	Recovered COVID-19 control_1 N = 75	Long COVID like symptoms no COVID-19 infection control_2 N = 44	No symptoms, no COVID-19 infection control_3 N = 89
Overall deficit score, median (25th, 75th quantiles) *	142	5 (2, 7)	3 (1, 4)	4 (3, 6)	4 (2, 7)
Autonomic domain, N (%)	210				
no deficits		44 (57.9%)	15 (65.2%)	33 (57.9%)	32 (59.3%)
any deficits		32 (42.1%)	8 (34.8%)	24 (42.1%)	22 (40.7%)
Brain domain, N (%)	306				
no deficits		85 (66.9%)	26 (68.4%)	49 (62.8%)	45 (71.4%)
any deficits		42 (33.1%)	12 (31.6%)	29 (37.2%)	18 (28.6%)
Exercise domain, N (%)	282				
no deficits		65 (60.7%)	17 (47.2%)	40 (51.9%)	43 (69.4%)
any deficits		42 (39.3%)	19 (52.8%)	37 (48.1%)	19 (30.6%)
Heart domain, N (%)	278				
no deficits		86 (76.8%)	26 (72.2%)	53 (76.8%)	47 (77.0%)
any deficits		26 (23.2%)	10 (27.8%)	16 (23.2%)	14 (23.0%)
Lung domain, N (%)	270				
no deficits		96 (89.7%)	27 (84.4%)	60 (87.0%)	51 (82.3%)
any deficits		11 (10.3%)	5 (15.6%)	9 (13.0%)	11 (17.7%)

Physical function domain, N (%)	328				
no deficits		72 (55.0%)	23 (54.8%)	50 (60.2%)	42 (58.3%)
any deficits		59 (45.0%)	19 (45.2%)	33 (39.8%)	30 (41.7%)
Renal domain, N (%)	287				
no deficits		92 (79.3%)	29 (82.9%)	65 (87.8%)	50 (80.6%)
any deficits		24 (20.7%)	19 (45.2%)	9 (12.2%)	12 (19.4%)
Strength domain, N (%)	333				
no deficits		89 (67.9%)	29 (70.7%)	60 (69.0%)	56 (75.7%)
any deficits		42 (32.1%)	12 (29.3%)	27 (31.0%)	18 (24.3%)
Vascular domain, N (%)	349				
no deficits		64 (45.4%)	19 (43.2%)	52 (58.4%)	34 (45.3%)
any deficits		77 (54.6%)	25 (56.8%)	37 (41.6%)	41 (54.7%)

* from a total of 27 potential deficits

Supplementary table S4 – Difference in overall deficit score and domain specific odds ratios between cases and controls – excluding those with missing values (complete case analysis, N=82)

	Model 1 beta, (95% CI)	Model 2 beta, (95% CI)	Model 3 beta, (95% CI)
**Difference in overall deficit score**	0.25 (-0.82,1.33)	0.24 (-0.87,1.35)	0.85 (-0.24,1.93)
**Domain specific odds ratio**			
**Autonomic**	1.31 (0.62,2.77)	1.28 (0.55,2.95)	1.33 (0.57,3.12)
**Brain**	1.44 (0.73,2.85)	1.42 (0.71,2.84)	1.43 (0.71,2.89)
**Exercise**	0.60 (0.31,1.15)	0.53 (0.26,1.08)	0.52 (0.25,1.08)
**Heart**	1.09 (0.52,2.30)	1.07 (0.51,2.22)	1.15 (0.55,2.41)
**Lung**	0.43 (0.17,1.10)	0.46 (0.18,1.18)	0.51 (0.19,1.37)
**Physical**	0.76 (0.38,1.51)	0.84 (0.39,1.80)	0.96 (0.44,2.09)
**Renal**	1.27 (0.58,2.82)	1.34 (0.58,3.13)	1.38 (0.60,3.21)
**Strength**	0.97 (0.55,1.72)	0.93 (0.52,1.66)	0.87 (0.48,1.58)
**Vascular**	1.50 (0.85,2.66)	1.47 (0.79,2.73)	1.75 (0.92,3.33)

Supplementary table S5 – Difference in overall deficit score and domain specific odds ratios between cases and controls – restricting cases to those reporting any symptoms at clinic (97/141)

	Model 1 beta, (95% CI)	Model 2 beta, (95% CI)	Model 3 beta, (95% CI)
**Difference in overall deficit score**	0.37 (-0.39,1.06)	0.31 (-0.40,1.02)	0.41 (-0.31,1.13)
**Domain specific odds ratios**			
**Autonomic**	0.81 (0.46,1.41)	0.79 (0.44,1.40)	0.80 (0.45, 1.44)
**Brain**	0.98 (0.54,1.77)	0.95 (0.52,1.73)	0.96 (0.52,1.75)
**Exercise**	0.95 (0.54,1.77)	0.92 (0.53,1.63)	0.92 (0.52, 1.75)
**Heart**	0.96 (0.51,1.84)	0.98 (0.51,1.88)	1.01 (0.52,1.95)
**Lung**	0.50 (0.20,1.26)	0.51 (0.20,1.34)	0.52 (0.20,1.40)
**Physical**	1.34 (0.76,2.34)	1.30 (0.73,2.33)	1.38 (0.77,2.49)
**Renal**	1.23 (0.66,2.28)	1.26 (0.66,2.41)	1.27 (0.66,2.42)
**Strength**	0.92 (0.54,1.56)	0.89 (0.52,1.52)	0.83 (0.48,1.44)
**Vascular**	1.80 (1.06,3.04)	1.69 (0.97,2.95)	1.97 (1.10,3.52)

Supplementary table S6 – Difference in overall deficit score and domain specific odds ratios between cases and controls – restricting cases to those reporting symptoms at least one of which was fatigue at clinic (46/141)

	Model 1 beta, (95% CI)	Model 2 beta, (95% CI)	Model 3 beta, (95% CI)
**Difference in overall deficit score**	0.75 (-0.23,1.73)	0.65 (-0.32,1.62)	0.69 (-0.27,1.66)
**Domain specific odds ratios**			
**Autonomic**	0.80 (0.39,1.64)	0.75 (0.35,1.60)	0.75 (0.36,1.60)
**Brain**	0.87 (0.39,1.94)	0.81 (0.36,1.84)	0.81 (0.35,1.86)
**Exercise**	1.22 (0.61,2.45)	1.19 (0.57,2.46)	1.18 (0.57, 2.47)
**Heart**	1.01 (0.43,2.38)	1.02 (0.44,2.40)	1.02 (0.43,2.41)
**Lung**	0.56 (0.14,2.29)	0.61 (0.15,2.57)	0.61 (0.15,2.60)
**Physical**	1.27 (0.60,2.67)	1.18 (0.54,2.54)	1.17 (0.54,2.51)
**Renal**	1.12 (0.51,2.43)	1.20 (0.52,2.76)	1.21 (0.52,2.77)
**Strength**	0.84 (0.40,1.78)	0.77 (0.35,1.68)	0.75 (0.33,1.70)
**Vascular**	3.02 (1.46,6.26)	2.83 (1.27,6.28)	3.04 (1.36,6.80)

Supplementary table S7 – Difference in overall deficit score and domain specific odds ratios between cases and controls – excluding 44 controls who had symptoms at clinic

	Model 1 (beta, 95% CI)	Model 2 (beta, 95% CI)	Model 3 (beta, 95% CI)
**Difference in overall deficit score**	0.32 (-0.37,1.01)	0.28 (-0.40,0.96)	0.45 (-0.24,1.14)
**Domain specific odds ratios**			
**Autonomic**	0.80 (0.47,1.35)	0.79 (0.46,1.36)	0.82 (0.47,1.41)
**Brain**	0.92 (0.53,1.62)	0.91 (0.51,1.61)	0.92 (0.52,1.65)
**Exercise**	0.92 (0.55,1.55)	0.89 (0.53,1.51)	0.89 (0.52,1.53)
**Heart**	1.00 (0.55,1.81)	1.04 (0.57,1.90)	1.07 (0.58,1.97)
**Lung**	0.76 (0.37,1.55)	0.76 (0.37,1.57)	0.82 (0.39,1.74)
**Physical**	1.39 (0.82,2.35)	1.36 (0.77,2.38)	1.54 (0.86,2.79)
**Renal**	1.20 (0.66,2.18)	1.27 (0.67,2.40)	1.28 (0.67,2.45)
**Strength**	1.08 (0.68,1.70)	1.02 (0.64,1.63)	0.93 (0.57,1.51)
**Vascular**	1.66 (1.02,2.69)	1.58 (0.94,2.67)	1.94 (1.10,3.42)

Supplementary Figure S1 – Flow chart of participation.

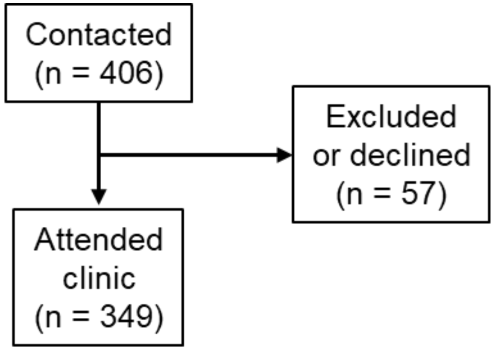

Supplement: Supplementary Fig. S1 and Tables S1–S7 [file mmc1.pdf]
