# Supplementary material for: Multi-organ structural and functional deficits in association with long COVID: a population-based case-control study
Source: eClinicalMedicine. 2026 Jul 23;98:104104. doi: 10.1016/j.eclinm.2026.104104 (PMC13427505; doi:10.1016/j.eclinm.2026.104104)
Supplement: Supplementary_list_of CONVALESCENCE_collaborative [file mmc2.docx]

**Names of all members of the CONVALESCENCE study collaborative (listed alphabetically).**

Lamia Al Saikhan

Frederick Barkhof

Gabriella Captur

Alisia Carnemolla

Nish Chaturvedi

Nathan Cheetham

Richard Dobson

Alba Fernández-Sanlés

Lucy J Goudswaard

Rebecca Green

Lee Hamill Howes

Vedika Handa

Alun Hughes

Alexandra Jamieson

Siana Jones

Rebecca Lewis

Vivek Muthurangu

Stefan Neubauer

Mary Ní Lochlainn

Michele Orini

Chloe Park

Praveetha Patalay

Stefan Piechnik

Maria Popham

Betty Raman

Alicja Rapala

Natalia Rojas

Laura C Saunders

Roz Shafran

Stephen M Smith

Terence Stephenson

Claire J Steves

Carole H. Sudre

Ellen J Thompson

Nicholas J Timpson

Thomas Triebel

Alex Whitmarsh

Jim Wild

Dylan M Williams

Andrew Wong
